# Comprehensive Report of the Caseload of Donkeys and Mules Presented to a Veterinary Medical Teaching Hospital over a Ten-Year Period

**DOI:** 10.3390/ani9070413

**Published:** 2019-07-03

**Authors:** Lais R. R. Costa, Monica Aleman, Eric Davis

**Affiliations:** Department of Medicine and Epidemiology, School of Veterinary Medicine, University of California, Davis, CA 95616, USA

**Keywords:** welfare, equids, ass, asses, *Equus asinus*, Equidae, mule

## Abstract

**Simple Summary:**

We sought to provide a comprehensive report of the caseload of donkeys and mules at the Veterinary Medical Teaching Hospital at the University of California, Davis for a ten-year period. The caseload of donkeys and mules was 1.06% of the total equid caseload and was comprised of animals that received a good standard of veterinary care, including both routine wellness and medical care. Most animals tended to be older, around 10 years of age, and possessed high body condition scores. Obesity was reported, especially in donkeys. Most mules were used for riding, packing or driving, and most donkeys were in sanctuary and rescue farms. We report in detail the preventative health and veterinary medical care of these donkeys and mules. Our findings provide a greater understanding of the health and welfare of donkeys and mules residing in the western United States.

**Abstract:**

Comprehensive reports of the caseload of donkeys and mules in veterinary hospitals in the United States are lacking. We compiled the information of the caseload of donkeys and mules at the Veterinary Medical Teaching Hospital at the University of California, Davis for a ten-year period, from 2008 to 2017. The overall equid caseload was 94,147, of which 996 (1.06%) were donkeys and mules. Most of the neonates seen were mules. Most miniature donkeys were between 2 and 10 years of age, and standard donkeys and mules were 10 to 20 years old. The body condition scores were predominantly high, especially in donkeys. Most miniature and standard donkeys resided in sanctuary and rescue farms and their use was not stated. Most mules were used for riding, packing or driving. Medical complaints represented 62% of the total visits and wellness visits represented 38% of total visits. The donkeys and mules in the case population described here received a good standard of veterinary care with regular vaccinations, deworming, routine dental care, and treatment of ailments. Our study is the first report of the life expectancy, use, body condition, preventative health and veterinary medical care of a population of donkeys and mules in the western United States.

## 1. Introduction

Donkeys and their hybrid crosses (here referred to as mules) have been working animals throughout human history, and have been often referred to as “beasts of burden”. These working animals are still crucial for rural economies in developing countries, where their populations are concentrated [1,2,3]. The largest number of reports in welfare and health issues affecting donkeys and mules have originated from the areas where these animals are most populous. Development in many parts of the world has meant that working equids were no longer needed, thus their population has experienced a tremendous decline [1,2]. A number of new or rediscovered uses of donkeys and mules in developed countries have evolved, including for companionship, outdoor leisure and performance activities, tourism packing and driving, guard animals, milk for cheeses and beauty products, meat for human and animal consumption, and hide byproducts [2,4,5,6]. Despite the diminished numbers of donkeys and mules in developed countries, their populations remain sizeable. Currently, the populations of donkeys and mules in the European Union are estimated to be around 288,000 and 160,000, respectively [3]. The populations of donkeys and mules for the United States and Canada was estimated to be around 52,000 and 28,000, respectively [3]. 

Reports addressing the veterinary care provided to donkeys and mules, including routine vaccination, deworming, dental care in addition to treatments of ailments are scarce or difficult to find. Mostly, reports address the types of ailments affecting donkeys and mules, often reflecting the animal’s age, sex, breed, use, and “life style” (including nutrition, workload). We sought to provide a comprehensive report of the caseload of donkeys and mules in a veterinary hospital in United States, with an emphasis on the animals, the reason for the visits, and the veterinary services. To our knowledge, this information has not been published. 

The aim of this study was to compile the information concerning the caseload of donkeys and mules, seen by the services of the Large Animal Clinic at the Veterinary Medical Teaching Hospital of University of California, Davis, for a ten-year period (1 January 2008 to 31 December 2017). Detailed information of the caseload is presented, with an overall emphasis on the patients’ signalment, body condition, use, the reason for the visit, and the service providing the care. 

## 2. Materials and Methods

The electronic medical record database for the Veterinary Medical Teaching Hospital at the University of California, Davis was searched for a period of 10 years from 1 January 2008 to 31 December 2017 for all donkey and mule cases seen at the Large Animal Clinic. The visit types at the Large Animal Clinic included: Large Animal Ambulatory Services, Large Animal Inpatient and Large Animal Outpatient. The overall and yearly caseload of the total equid population presenting to the Large Animal Clinic for the same period was compared to the caseload of donkeys and mules. 

Individual medical records of donkeys and mules in the electronic medical database were reviewed and information from each visit was collected. For this study, the terms cases and visits are used interchangeably. Wellness visits are defined as visits where the animals received any routine health examinations, and at least one routine procedure. Routine health examinations included annual heath examinations, health certificates, neonatal wellness exams, post-partum exams, geriatric wellness exams, entrance exams, breeding soundness exams, pregnancy checks and pre-purchase exams. Routine procedures included vaccinations, deworming, dental care, routine castration, sedation for foot care or sheath cleaning, and blood sample collection for Coggin’s test and routine blood work, and fecal sample for egg counts. The information collected from the medical records included: the signalment (age, sex) and use, body condition score (BCS), reason for visit (either wellness visit or presenting complaint) and veterinary service provided, visit type (outpatient, versus inpatient or hospitalized), number of days of hospitalization, clinical diagnosis, and discharge status. Medical and surgical complaint visits were categorized by the body systems involved. The discharge status was listed as live, euthanized, and dead, where dead indicates animals that died without euthanasia. 

The data of the study were summarized and reported as descriptive statistics using GraphPad Prism version 8 for Windows, GraphPad Software, La Jolla, CA, USA.

## 3. Results

### 3.1. Patient Population Statistics

**Figure 1:**
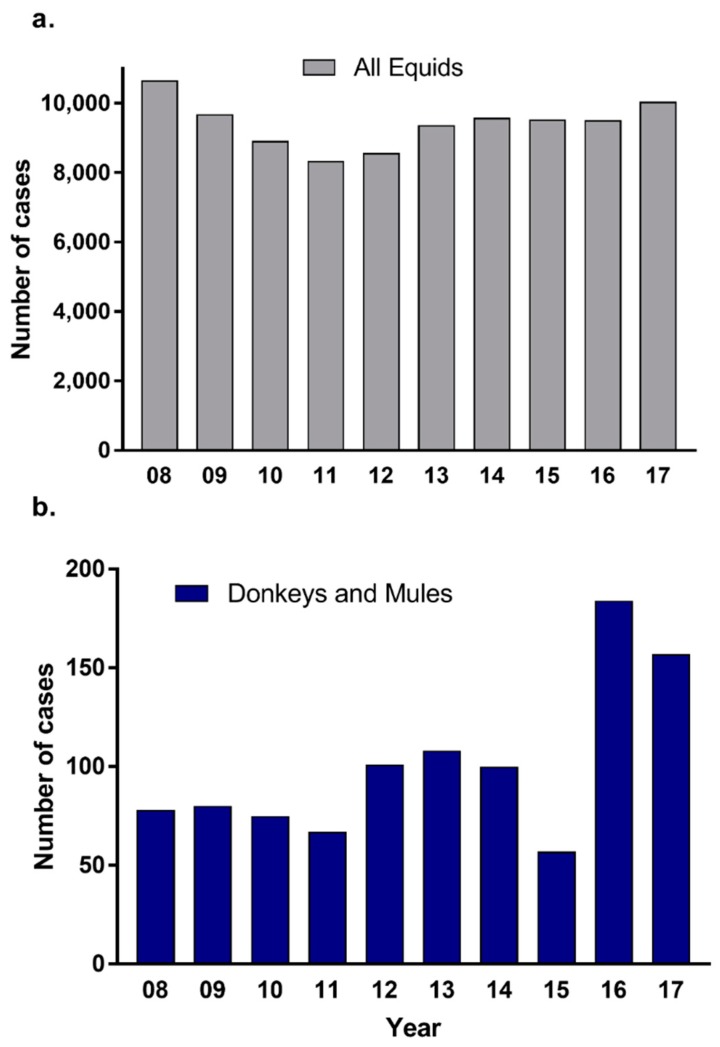
Distribution of the Visits of Equids for Each Year from 2008 to 2017. The yearly caseload for: (**a**) all equids (grey), and (**b**) donkeys and mules combined (blue).

For the 10 years period between 1 January 2008 to 31 December, 2017 at the Large Animal Clinic, the overall equid caseload was 94,147, of which 996 were donkeys and mules (1.06%). The overall yearly mean and standard deviation of the equid caseload was 9415 ± 682, ranging from 8561 to 10,652 cases per year (Figure 1a). The yearly mean and standard deviation of the caseload of donkeys and mules was 101 ± 40, ranging from 58 to 184 cases per year (Figure 1b).

The caseload of donkeys and mules at the UC Davis Veterinary Medical Teaching Hospital (VMTH) over a 10-year period of 1.06% of the total equid caseload is proportionally slightly higher than the overall estimated proportion of donkey and mule populations in the United States (Table S1). Most visits were donkeys (0.61%) as compared to mules (0.45%), which is congruent with the estimated distribution within the United States.

**Figure 2:**
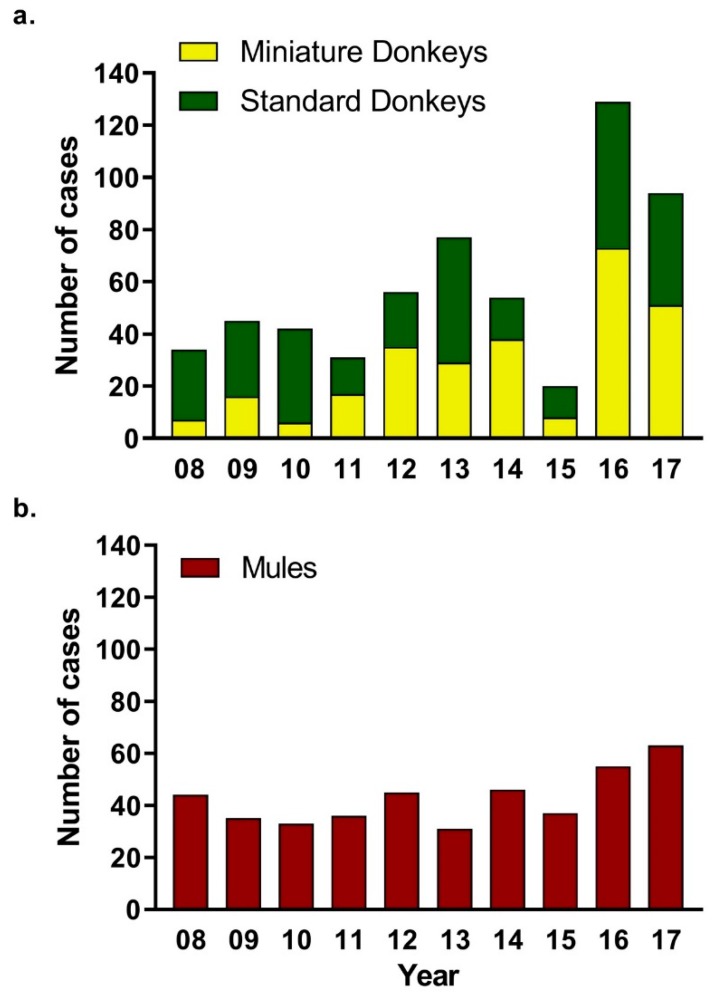
Distribution of the Donkey and Mule Visits for Each Year, from 2008 to 2017. The yearly distribution of the caseload for: (**a**) donkeys, including standard donkeys (green), miniature donkeys (yellow), and (**b**) mules (red).

The yearly caseload distribution of donkeys fluctuated more over the years when compared to the caseload distribution of mules (Figure 2). The caseload of miniature donkeys increased from less than 10% in 2008 to nearly 40% in 2016.

Out of the 996 total donkey and mule visits, a total of 575 visits were donkeys (with 312 visits being standard donkeys, and 263 being miniature donkeys) and 421 were mules. Several animals were seen more than once a year or by more than one service.

**Figure 3:**
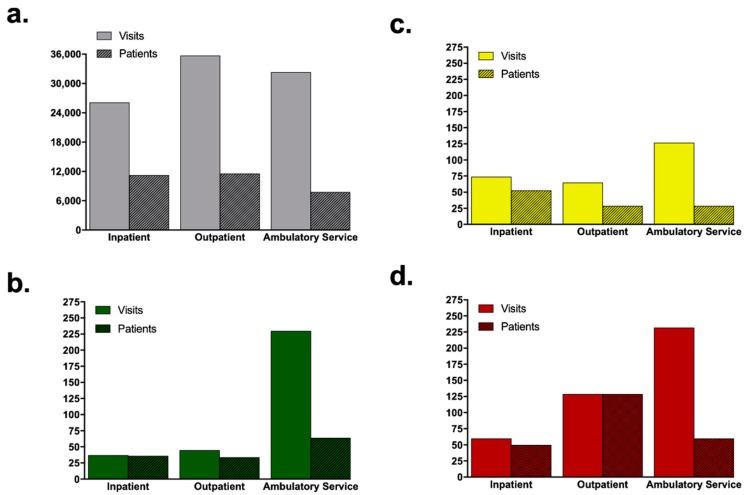
Distribution of the Visits and Patients by Visit Type in the Large Animal Clinic. The X-axis shows the three visit types: Ambulatory Service, Outpatient and Inpatient; Visits are depicted in solid color and patients are in color with pattern: (**a**) All equids (grey), (**b**) standard donkeys (green), (**c**) miniature donkeys (yellow), and (**d**) mules (red).

The caseload of all equids at the Large Animal Clinic was categorized according to the three visit types into: Large Animal Outpatient visits (day cases seen at the VMTH), Large Animal Inpatient (cases hospitalized at the VMTH), and Large Animal Ambulatory Service visits (cases seen by the ambulatory services on farm calls). The caseload when categorized according to the visit type revealed that for the overall equid population the highest visit type was Large Animal Outpatient visits (35,695/94,147), closely followed by Large Animal Ambulatory Service visits (32,310/94,147) (Figure 3, Table 1). Most donkey and mule visits were Large Animal Ambulatory Service visits (587/996). Of the 587 Large Animal Ambulatory Service visits, 230 were standard donkeys (39%), 125 were miniature donkeys (21%) and 232 were mules (40%) (Figure 3, Table 1). 

The ratio of number of visits per patient was calculated for each visit type (Table 1). The ratio of number of visits per patient was highest for the Ambulatory Service visits, for all equids (4.2), as well as for standard donkeys (3.6), miniature donkeys (4.3) and mules (3.9). Mules had the lowest ratio of number of visits per patient for all three visit types.

Donkey cases presenting to the Large Animal Clinic as inpatients came mostly from northern and central California, and a few came from as far away as the Los Angeles basin and Death Valley in southern California, Reno Nevada, and Portland Oregon. Donkey cases presenting to the Large Animal Clinic as outpatients came mostly from northern California and a few came from central California. Mule cases seen as inpatients and outpatient resided in northern and central California, and a few in Nevada. Donkey and mule cases seen by the Ambulatory Services were concentrated in the southern Sacramento valley within a 50-mile radius around Davis. The locations where the donkeys and mules resided are depicted in Figures S1–S6. Cases presenting as inpatients and outpatients were often referral cases that had been seen by a primary care practitioner. The Ambulatory Services constitute one of the primary care services. 

### 3.2. Donkey and Mule: Visits and Patients

#### 3.2.1. Ages of Donkeys and Mules

**Figure 4:**
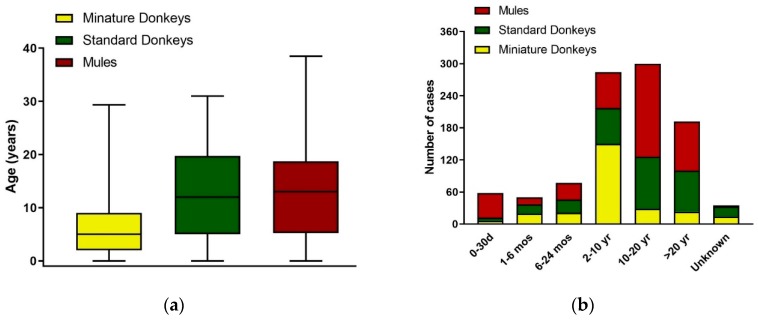
Age Distribution of Donkey and Mule Cases from 2008 to 2017. (**a**) Age distribution by median, interquartile and range; (**b**) Age distribution by categories: 0 to 30 days, greater than 1 month to 6 months, greater than 6 months to 24 months, greater than 2 years to 10 years, greater than 10 years to 20 years, and greater than 20 years; d = days; mos = months; yr = years. Standard donkeys (green), miniature donkeys (yellow), and mules (red).

With respect to their ages, the mean and standard deviations of ages for standard donkeys was 12.3 ± 8.6 years (range 0 to 31), for miniature donkeys was 7.2 ± 7.3 years (range 0 to 29) and for mules was 12.5 ± 8.9 years (range 0 to 38.5). The median, interquartile and range of ages for standard donkeys, miniature donkeys and mules are depicted in Figure 4a. There were 35 medical records (3.5%) that did not report the age of the animal. Of the 35 medical records where age was not reported, 19/312 were standard donkeys, 14/263 were miniature donkeys and 2/421 were mules. These cases were all adult animals and they were not included in the statistical calculations. Due to the wide range of ages, the ages of the animals were grouped into categories: 0 to 30 days, greater than 1 month to 6 months, greater than 6 months to 24 months, greater than 2 years to 10 years, greater than 10 years to 20 years, and greater than 20 years. The distributions of age categories of the donkeys and mules are depicted in Figure 4b. For miniature donkeys the highest frequency category was 2 to 10 years of age (150/263), whereas for standard donkeys (97/312) and mules (174/421) the highest frequency category was 10 to 20 years of age. Most of the age group 0 to 30 days were mule neonates (46/421). 

#### 3.2.2. Sex of Donkeys and Mules

**Figure 5:**
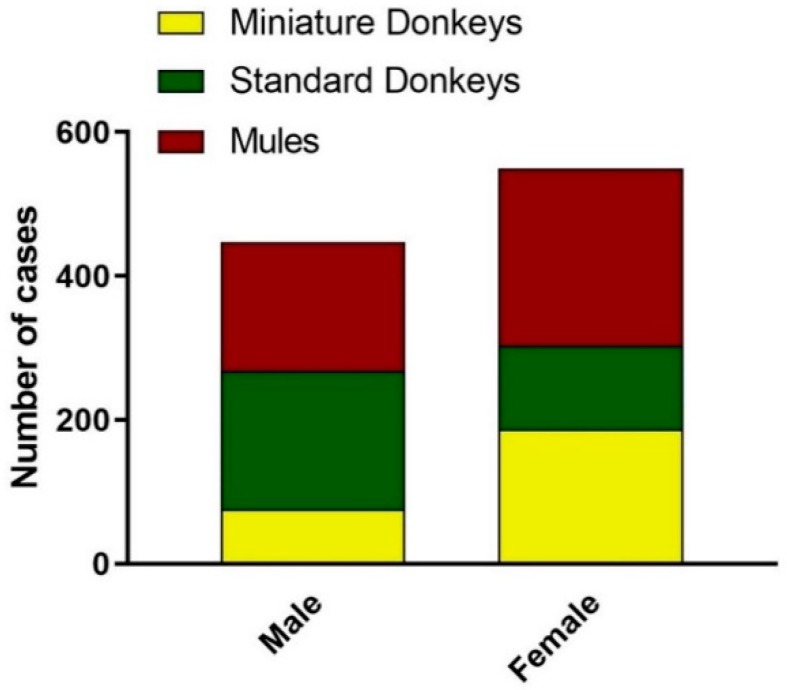
Sex Distribution of Donkey and Mule Caseload from 2008 to 2017. Standard donkeys (green), miniature donkeys (yellow), and mules (red).

There was an overall predominance of females, due to the predominance of mule and miniature donkey females. Standard donkeys had a predominance of males. The sex distribution is depicted in Figure 5.

#### 3.2.3. Use of Donkeys and Mules

**Figure 6:**
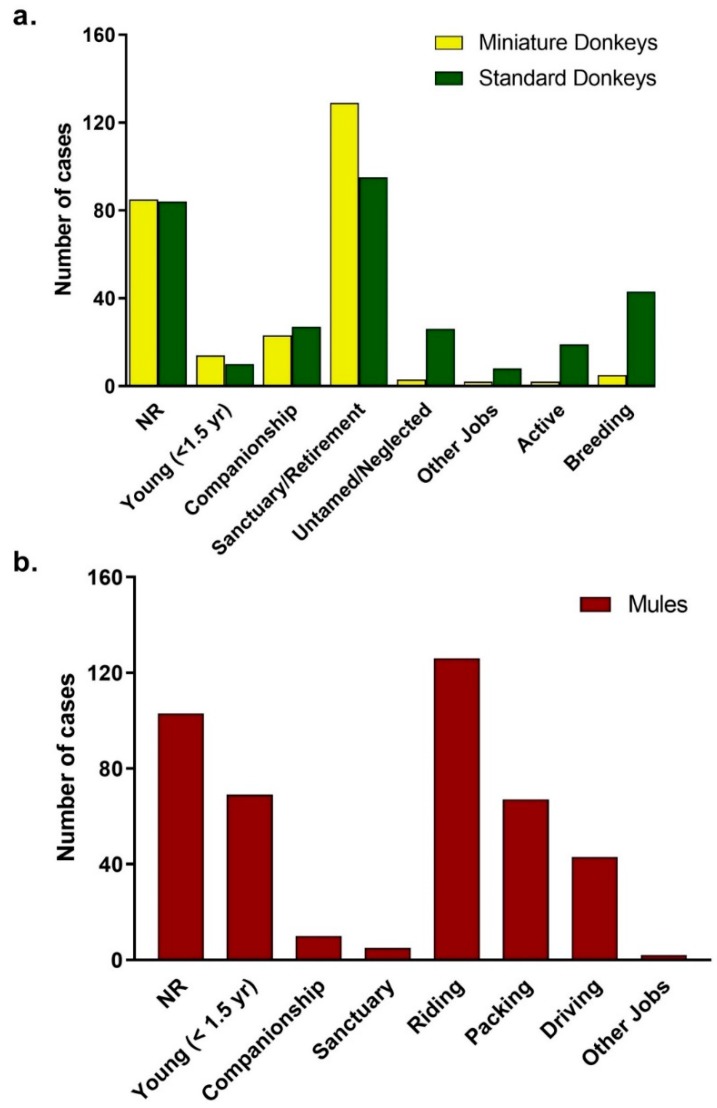
Distribution of Use for Donkey (**a**) and Mule (**b**) Caseload from 2008 to 2017. NR = Not reported in the medical record. Standard donkeys (green), miniature donkeys (yellow), and mules (red).

Overall, the information characterizing the use of the animal was reported in 73% of the cases. The use of the animal was not reported in 27% (84/312) of standard donkeys, and 32% (85/263) miniature donkeys, and 24% (103/421) of mules. The distribution of donkey and mule cases by use of the animal or “profession” is depicted in Figure 6. Many donkeys, especially the miniature donkeys, were in the category of “Sanctuary/Retirement”, which included animals in sanctuary, rescue farms, and retirement, as it appeared that these animals were not engaged in regular activities. Only 5 mules were in a similar category. Both donkeys and mules had “Companionship” category. For donkeys, the “Active” category included all animals used for riding at trail rides, and riding and driving at parades. For mules, the activity categories were listed individually; if an animal was used for more than one activity, the primary use was tallied. The “Other Jobs” category for donkeys included guard animals, blood donors and animals in petting zoos. For mules, the category of “Other Jobs” included animals used for teaching. Donkeys had a category of “Untamed or Neglected”, which included animals unhandled or untamed and those neglected and seized by Animal Control Services. The category “Breeding” included animals used for reproduction. Sanctuary and Companion were the highest categories for miniature donkeys (152/263 cases) and standard donkeys (122/312). Most mules were reported to be active and the categories: Driving (43/421), Riding (126/421) and Packing (67/421). There were 23/67 mules used for packing and riding.

#### 3.2.4. Body Condition Scores of Donkeys and Mules

**Figure 7:**
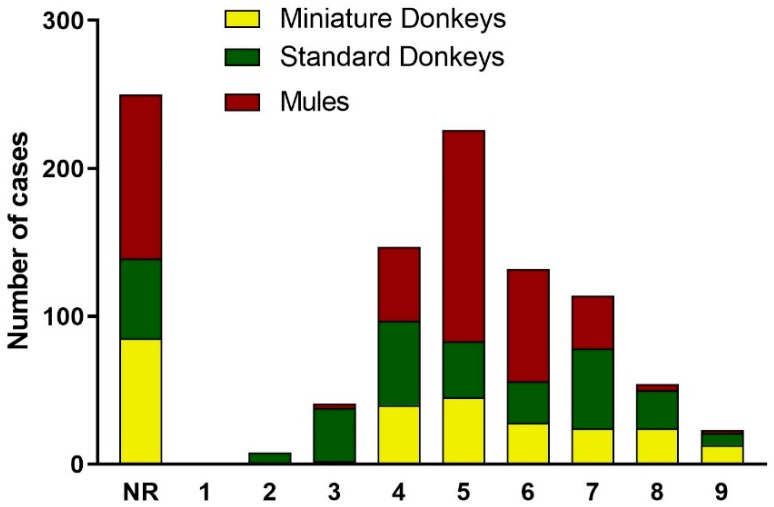
Distribution of the Body Condition Scores for Donkey and Mule Caseload from 2008 to 2017. NR = Not reported in the medical record. Standard donkeys (green), miniature donkeys (yellow), and mules (red).

The body condition scores (BCS) reported in the medical records used the scale 1 to 9 that is published for horses [7]. The distribution of BCS for standard donkeys, miniature donkeys and mules are depicted in Figure 7. The BCS was not recorded in 24% of the medical records, being 27% of the standard donkeys (85/312), 21% of the miniature donkeys (54/263), and 26% of the mules (111/421). When examining the data for each of the three animal categories, the distribution of the BCS for donkeys was skewed to the right (high BCS). The mode BCS for standard donkeys was 4/9 BCS, for mules and miniature donkeys was 5/9 BCS. Lower BCS (less than 4/9) was much less frequent, occurring 14% in standard donkeys (43/312), 1.1% in miniature donkeys (3/263), and 0.71% in mules (3/421). On the other end of the spectrum, 31% of standard donkeys (98/312), 23% of the miniature donkeys (61/263) and 10% of the mules (42/421) had BCS of 7/9 or greater. 

#### 3.2.5. Reason for the Visits of Donkeys and Mules

**Figure 8:**
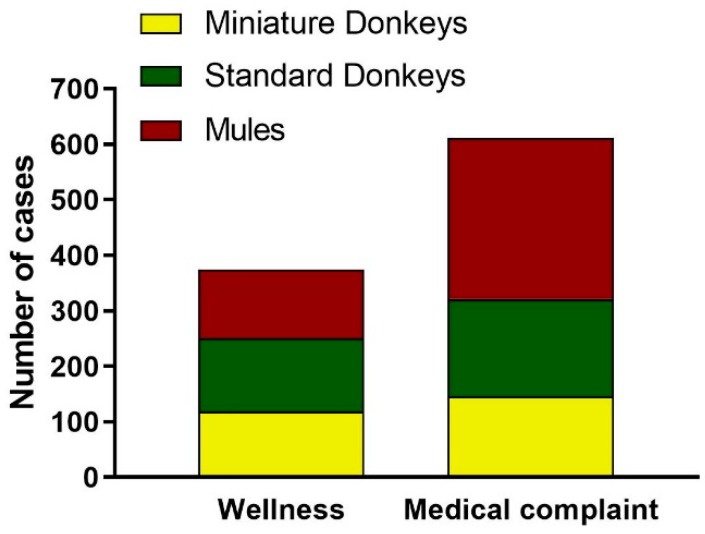
Distribution of Reason of the Visit for Donkey and Mule Caseload from 2008 to 2017. The distribution of donkey and mule visits divided into visit categories: Wellness Visits and Medical Complaint Visits. Standard donkeys (green), miniature donkeys (yellow), and mules (red).

The reason for the visits was divided into: Wellness visits and medical complaints visits. The number of visits for wellness and medical complaint were calculated for standard donkeys, miniature donkeys and mules and are graphically depicted in Figure 8.

Wellness visits comprised 38% (375/996) of all visits. Wellness visits included one of the wellness exams, and at least one of the routine procedures such as vaccinations, routine dental care, deworming, routine castration, sedation for foot care and sheath cleaning, sample collection for Coggin’s test, routine blood work and fecal egg counts. Frequently, more than one of the routine procedures were provided in one single visit. There was a total of 43 routine male castrations (22 miniature donkeys, 12 mules and 9 standard donkeys). A total of 203 wellness visits included routine vaccinations (72 standard donkeys, 70 miniature donkeys and 61 mules). Routine dental care was provided in 56 visits (35 mules, 14 standard donkeys and 7 miniature donkeys). There was a total of 17 reproductive wellness visits (which included pregnancy checks, breeding and soundness exams and post-partum exams), of which 9 were miniature donkeys and 8 were standard donkeys.

**Figure 9:**
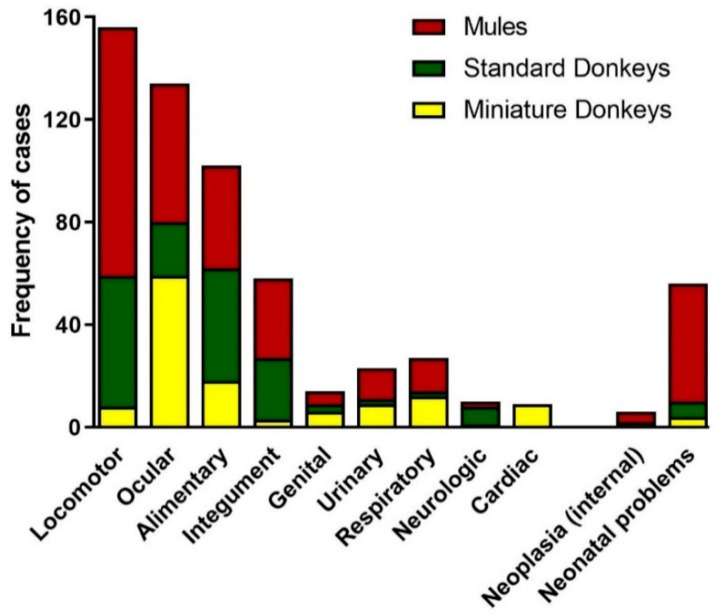
Distribution of Medical Complaint Visits of Donkeys and Mules from 2008 to 2017. The visits were categorized according to the primary body systems involved in the presenting medical complaint. Standard donkeys (green), miniature donkeys (yellow), and mules (red).

Medical complaints (622/996) represented 62% of the total visits. Most of the medical complaint visits were mules (301/622, 47%), followed by standard donkeys (176/622, 29%) and miniature donkeys (145/622, 23%). Overall, nearly 70% (301/421) of the mule visits, 56% (176/312) of the standard donkey visits and 55% (145/263) of the miniature donkey visits were categorized as medical complaint visits. Elective surgeries, which included ovariectomy and cryptorchid castration and excluded routine castrations, were compiled in the medical complaint visits. The overall medical complaint visits when categorized by body system revealed that the four most common body systems involved included locomotor (orthopedic and hoof conditions), ocular (all structures of the eye including eyelids), alimentary (dental and gastrointestinal conditions), and skin conditions (Figure 9).

#### 3.2.6. Equine Service Providing the Veterinary Care for Donkeys and Mules

**Figure 10:**
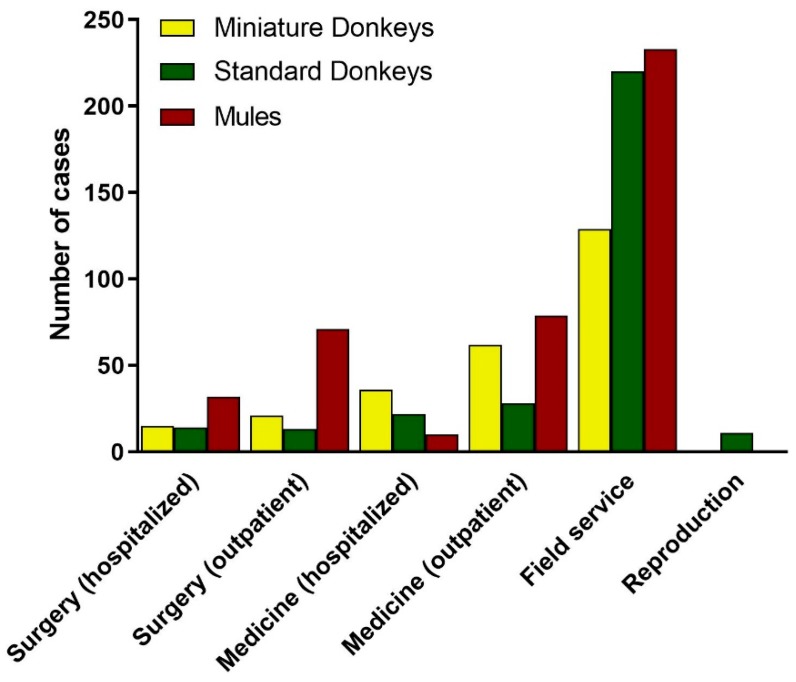
Distribution of Donkey and Mule Caseload from 2008 to 2017 across the Equine Services. Standard donkeys (green), miniature donkeys (yellow), and mules (red).

Considering the distribution of the donkey and mule caseload across the equine services, there was a predominance of visits provided by the Equine Field Service (59%, 589/996). Out of the Equine Field Service visits, 41% (240/589) were mules, 37% (220/589) were standard donkeys and 22% (129/589) were miniature donkeys (Figure 10). The majority of inhouse outpatient visits for both Medicine and Surgery Services were mules, followed by miniature donkeys. (Figure 10). In total, 58% (240/421) of the mule visits, 71% (220/312) of standard donkey visits and 49% (129/263) miniature donkey visits were provided by the Equine Field Service. 

The total number of hospitalizations was 127, being 51 miniature donkeys, 40 mules and 36 standard donkeys; and the length of hospitalization averaged 6 ± 4 days (ranging from 1 to 17 days) for miniature donkeys, 4 ± 3 days (ranging from 1 to 16 days) for mules, and 5 ± 3 days (ranging from 1 to 13 days) for standard donkeys. Overall, there were 67 hospitalizations in the Equine Medicine Service, which also included cases for ophthalmology, oncology and cardiology. The length of hospitalization in the Equine Medicine Service averaged 7 ± 4 days, ranging from 2 to 17 days for miniature donkeys (*n* = 36), 5 ± 4 days, ranging from 1 to 13 days for standard donkeys (*n* = 22), and 5 ± 4, days ranging from 2 to 16 days for mules (*n* = 9). There were 60 hospitalizations in the Equine Surgery Service, with the length of hospitalization in the Equine Surgery Service averaging 7 ± 2 days, ranging from 2 to 10 days for miniature donkeys (*n* = 15), 4 ± 2 days, ranging from 2 to 11 days for standard donkeys (*n* = 14), and 4 ± 3, days ranging from 2 to 12 days for mules (*n* = 31).

Some of the animals presented to the Large Animal Clinic to accompany the actual patient and were hospitalized as companions. Nearly 6% of the miniature donkeys (15/263) and 2% of the standard donkeys (6/312) were hospitalized as companions to the actual patients. No mules were hospitalized as companions to patients. 

With respect to discharge status of the 388 patients, 36 died. Seven of the 36 were miniature donkeys (4 of 7 were euthanized), 19 were standard donkeys (16 of 19 were euthanized), and 11 were mules (all were euthanized).

## 4. Discussion

The donkey and mule case population we described here received an overall good standard of care, where regular veterinary care in the form of vaccinations, deworming and routine dental care and treatment of ailments appeared to be a norm. The body condition scores and use of the animals described here indicate that malnourishment, starvation and overwork were not prevalent. Our study provides a better indication of the health status and management of donkeys and mules in our geographic area. It remains to be determined if the donkey and mule cases we reported here actually reflect the overall population in North America, since a similar study has not been published. 

Our results contrast with those reported in a three-year retrospective study of diseases affecting working equids in Jordan reporting a caseload of 1261 cases. Of the 1261 equids, most were horses, only 5.9% were donkeys and 1% were mules; while the estimated country equid population at the time had 71% donkeys, 18% horses and 11% mules [8]. The authors do not provide an explanation for this disparity, but suggested that donkeys and mules are thought to be tougher and they tend to be owned by people with a lower income. The study in Jordan presents only medical and surgical problems, many of which arise from inadequate nutrition and management, poor housing conditions, animal misuse and improper equipment such as harnesses and saddles. The retrospective study in Jordan does not report visits for preventative veterinary care, and indicates that routine health care such as vaccinations, deworming, routine dental care was not provided to these equids due lack of owner awareness and the cost, raising the concerns about the welfare of these working equids [8]. Similarly, other studies report high incidence of poor body condition scores, wounds due to poorly designed equipment such as harness and saddles, dental abnormalities, ocular conditions and lameness in working donkeys in several countries [9,10]. Although our study included animals receiving a good standard of heath care, the four body systems most commonly affected in the medical complaint visits (i.e., locomotor, alimentary, ocular and integument) were similar to those reported worldwide.

Our data reflects a number of very pertinent health and welfare issues, especially with respect to the age and life expectancy, use, body condition, and preventative and medical health care of the animals in the population evaluated in this study. Firstly, the overall age of the population of animals in our study highlights the fact that the animals tended to be older and even geriatric. The older demographic is likely related to the fact that many of these animals are either involved in light work or not working, in contrast with the much shorter life expectancy of working equids in developing countries. Veterinary care has greatly improved the life expectancy of working donkeys. A study in Mali between 2005 to 2009 reported that an average of 62% working donkeys died within 8 months of starting work. Among villages, the mortality rate varied from 33% to 100% for donkeys with less than 8 months of work. However, after implementation of veterinary care and owner education by SPANA (The Society for the Protection of Animals Abroad - an international charity for working animals), the mortality rate dropped to 5% and the average work life span extended to 5.25 years, ranging from 4 to 8 years after animals began working for the period of 2013 to 2017 [11]. 

The older demographic demonstrates that adequate husbandry and health care was being provided to the population of donkeys and mules seen at our clinic. These animals received proper nutrition, regular foot care, dental care, vaccination and deworming, which are all important aspects that affect the animals’ life span. Hand-in-hand with the age of the animals was the body condition scores of the animals in the population evaluated here, which tended to be much higher than that reported for working donkeys [9,10]. Some animals had very high BCS, indicating that obesity was a concern in this population of donkeys, especially miniature donkeys. Obesity and dyslipidemia in donkeys are of great concern, representing a serious medical disturbance of donkeys [12,13,14]. Most donkeys in our study were not used for work, and instead were residents of sanctuary and rescue farms. Mules were commonly used for light work, including packing, driving, riding and a combination of these activities, and not surprisingly, there were much fewer obese animals.

Although miniature and standard donkeys represent an increasing proportion of the equid populations presenting at the Veterinary Teaching Hospital at the University of California—Davis, they still represent a minority compared to the number of equids presenting at the hospital. The mule caseload appears to be largely stable at present. The data presented here demonstrates that in northern California, the use of donkeys, both standard and miniature, has shifted away from working. Although some donkeys are still used for light work, many are now used as companion animals or residing in sanctuary farms. Thus, the increasing number of donkey cases may reflect the increasing number of sanctuary farms in northern California. 

Sanctuaries are becoming more common as natural areas and range lands continue to diminish as a result of increasing urbanization and loss of open areas. Overpopulation of feral equids (horses, donkeys and mules), abandonment of unwanted animals, and animal abuse and neglect on government or public lands has been a significant problem over the past several decades. Agencies such as the Bureau of Land Management are moving equids from public lands into adoption programs and sanctuaries where they have access to husbandry and veterinary medical attention with the hope of fulfilling the five freedoms. The five freedoms are considered the gold standard of animal welfare and include freedom from hunger and thirst, discomfort, pain, injury, and disease, fear and distress, and the freedom to express normal and natural behavior. The benefits of fulfilling the five freedoms may outweigh the cost of no longer having free roaming equids across the West.

Our study was limited by the retrospective nature of the data collection and that some medical records were incomplete and did not include information about age, use and body condition score. Additionally, in all the medical records the body condition scores were recorded using a 1 to 9 scale, which is a scale used for horses, but it is not the scale recommended for donkeys. Although the recommended body scoring of donkeys uses the 1 to 5 scale, we believed that transforming the scores for the donkeys from the 1 to 9 scale into the 1 to 5 scale had the potential to introduce error. Thus, we maintained the body condition scoring as per the animals’ medical records, but we point out that the body scoring of donkeys is recommended to follow the 1 to 5 scale as published [12]. Despite this disparity in the body scoring scales, the take-home message of our study remains unchanged regardless of which body scoring scale system is used.

The data presented here provide a greater understanding of some of the factors affecting the welfare of a population of donkeys and mules residing in a part of western United States, and highlight the importance of these animals in our society.

## 5. Conclusions

The caseload of donkeys and mules at the UC Davis Veterinary Medical Teaching Hospital over a 10-year period was about 1.06% of the total equid caseload and was comprised of animals that received a good standard of veterinary care. Animals tended to be older and with high body condition scores with many cases of obesity. Most mules were used for riding, packing or driving, and most donkeys resided in sanctuary and rescue farms. This is the first publication that reports age and life expectancy, use, body condition, and preventative health and medical care of a population of donkeys and mules residing in the western United States.

## Figures and Tables

**Table 1:** Ratio of Number of Visits per Patient for Visit Types at the Large Animal Clinic.

Categories		LA Ambulatory	LA Outpatient	LA Inpatient	Total
**All Equids**	Visits (V)	32,310	35,695	26,142	94,147
	Patients (P)	7794	11,587	11,268	30,649
	V/P Ratio	4.1	3.1	2.3	3.07
**Standard Donkeys**	Visits (V)	230	45	37	312
	Patients (P)	64	34	36	134
	V/P Ratio	3.6	1.3	1.1	2.3
**Miniature Donkeys**	Visits (V)	125	64	74	263
	Patients (P)	29	29	53	111
	V/P Ratio	4.3	2.2	1.4	2.4
**Mules**	Visits (V)	232	129	60	421
	Patients (P)	60	129	50	239
	V/P Ratio	3.9	1.0	1.2	1.8

## Supplementary Materials

The following are available online at https://www.mdpi.com/2076-2615/9/7/413/s1, Figure S1: Donkey Inpatient Cases: northern California (including north coast, bay area, Sacramento valley, Sierra foothills east of the southern Sacramento) and central California (the San Joaquin valley, Sierra foothills east of San Joaquin valley and central coast), Los Angeles basin and Death Valley in southern California, Reno area in Nevada, and Portland area in Oregon, Figure S2: Donkey Outpatient Cases: northern California (including north coast, bay area, Sacramento valley, northern San Joaquin valley, Sierra foothills and east of the southern Sacramento), and Central part of California (including San Joaquin valley and the central coast areas), Figure S3: Donkey Ambulatory Service Cases: all concentrated in the southern Sacramento valley within a 50-mile radius around Davis, Figure S4: Mule Inpatient Cases: northern California (including Trinity/ Shasta alps, north coast, Sacramento valley), central California (including San Joaquin valley, Sierra Nevada foothills and southern Sierra and central coast), and Reno area in Nevada, Figure S5: Mule Outpatient Cases: northern California (including north coast, Sacramento valley), central California (including San Joaquin valley, Sierra Nevada foothills and southern Sierra and central coast), and Reno area in Nevada, Figure S6: Mule Ambulatory Service Cases: all concentrated in the southern Sacramento valley within a 50-mile radius around Davis, Table S1: Summary of the number of equids, with breakdown into donkeys, horses and mules.
