# Supplementary material for: Comprehensive Report of the Caseload of Donkeys and Mules Presented to a Veterinary Medical Teaching Hospital over a Ten-Year Period
Source: Animals (Basel). 2019 Jul 3;9(7):413. doi: 10.3390/ani9070413 (PMC6680720; doi:10.3390/ani9070413)
Supplement: Supplementary file 1 [file animals-09-00413-s001.pdf]

animals

MDPI

Supplementary Materials: Comprehensive Report of the Caseload of Donkeys and Mules Presented to a Veterinary Medical Teaching Hospital over a Ten-Year Period

Lais R. R. Costa *, Monica Aleman and Eric Davis

Table S1. Summary of the number of equids, with breakdown into donkeys, horses and mules.

Year	Location	Donkeys	Horses	Mules	Total Equids	% Donkey	% Mule
**2017**	**World ***	45,798,365	60,566,601	9,685,278	116,050,244	39.464	8.346
**2017**	**USA ***	51,975	10,510,748	0; no data	10,562,723	0.494	0; no data
**2005**	**USA ***	52,000	9,200,000	28,000	9,280,000	0.560	0.302
**2008–2017**	**VMTH Visits**	575	93,693	421	94,147	0.61	0.45

* FAOSTAT [1].

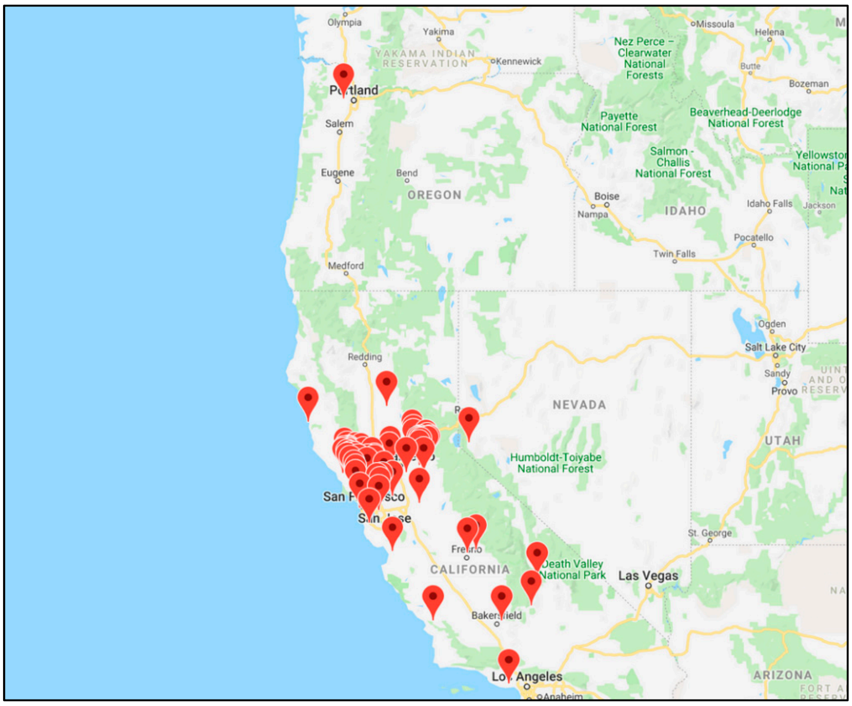

Figure S1. Donkey Inpatient Cases: northern California (including north coast, bay area, Sacramento valley, Sierra foothills east of the southern Sacramento) and central California (the San Joaquin valley, Sierra foothills east of San Joaquin valley and central coast), Los Angeles basin and Death Valley in southern California, Reno area in Nevada, and Portland area in Oregon.

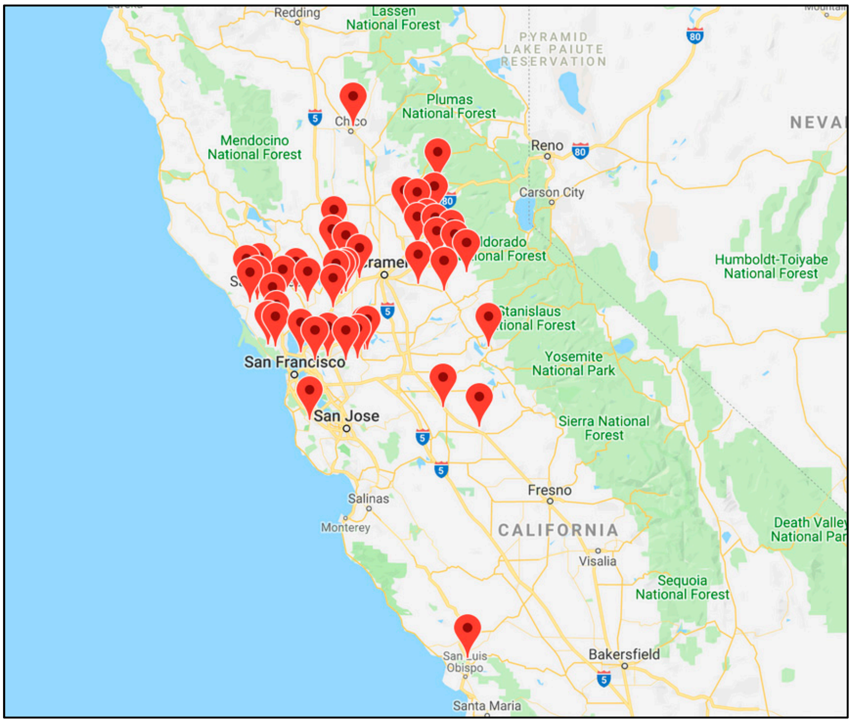

Figure S2. Donkey Outpatient Cases: northern California (including north coast, bay area, Sacramento valley, northern San Joaquin valley, Sierra foothills and east of the southern Sacramento), and Central part of California (including San Joaquin valley and the central coast areas).

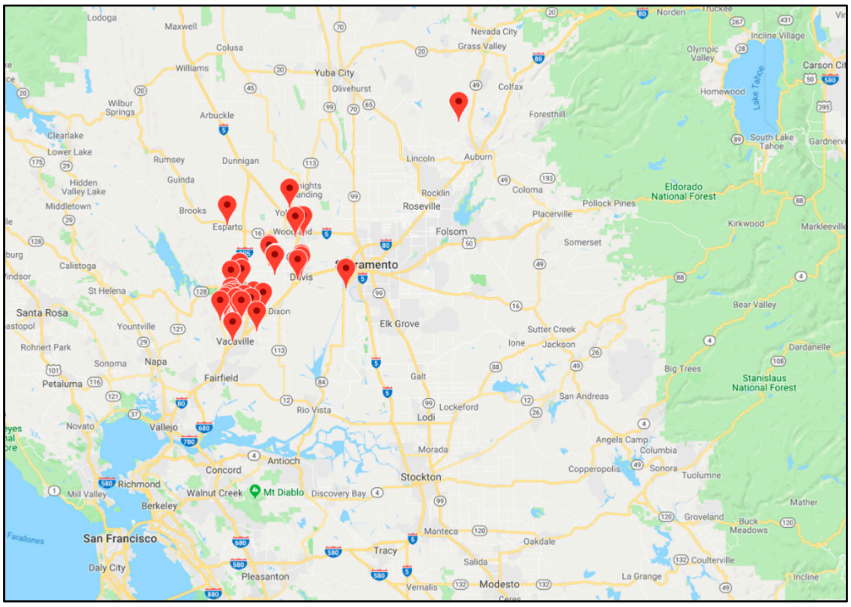

Figure S3. Donkey Ambulatory Service Cases: all concentrated in the southern Sacramento valley within a 50-mile radius around Davis.

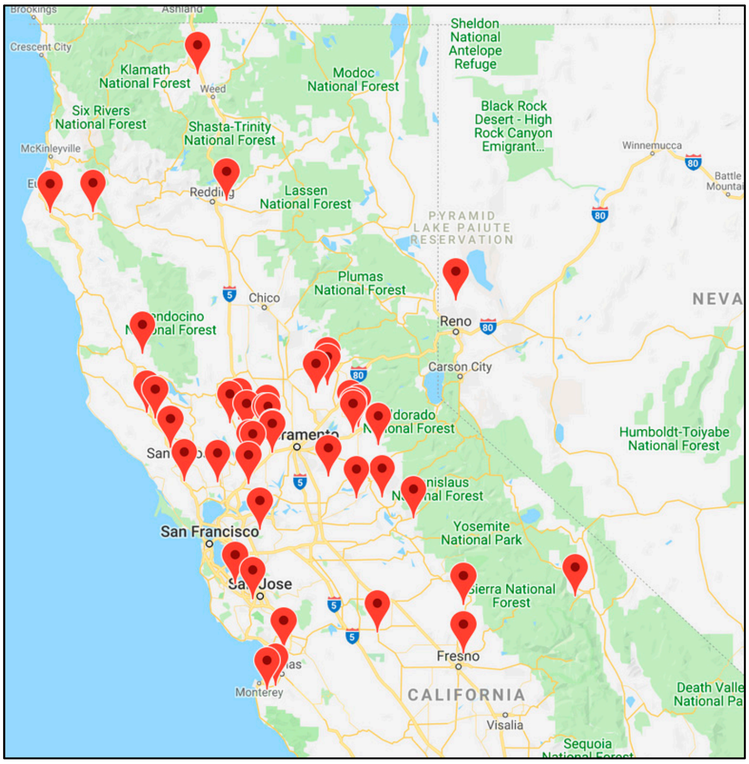

Figure S4. Mule Inpatient Cases: northern California (including Trinity/ Shasta alps, north coast, Sacramento valley), central California (including San Joaquin valley, Sierra Nevada foothills and southern Sierra and central coast), and Reno area in Nevada.

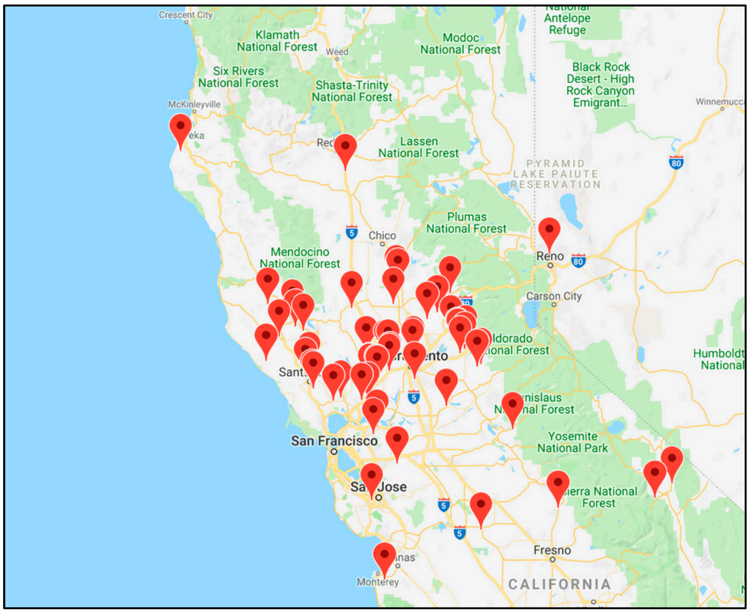

Figure S5. Mule Outpatient Cases: northern California (including north coast, Sacramento valley), central California (including San Joaquin valley, Sierra Nevada foothills and southern Sierra and central coast), and Reno area in Nevada.

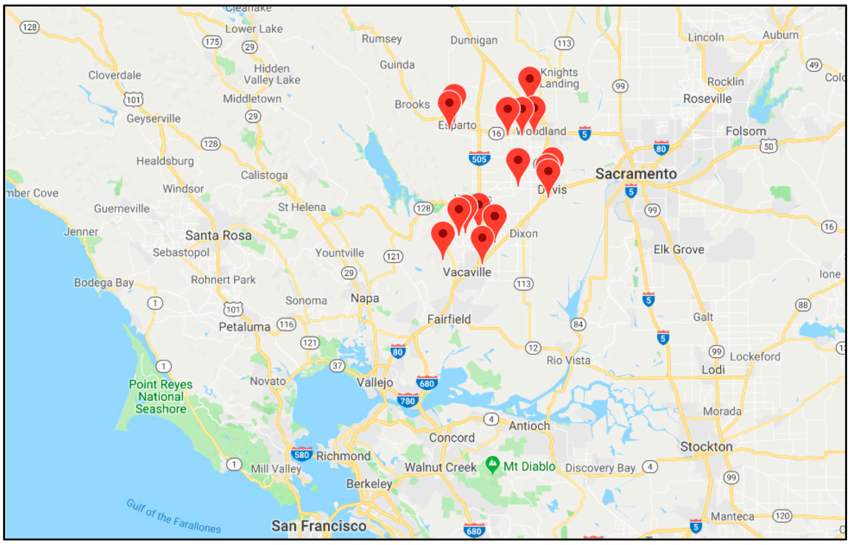

Figure S6. Mule Ambulatory Service Cases: all concentrated in the southern Sacramento valley within a 50-mile radius around Davis.

References

1. FAOSTAT. Available online: http://www.fao.org/faostat/en/#home (accessed on 2 July 2019).
